# Febrile infection-related epilepsy syndrome (FIRES) in adults: a case report and review of factors associated with survival

**DOI:** 10.1007/s10072-025-08728-0

**Published:** 2026-01-12

**Authors:** Zhibin Tan, Hua Chan Ling, You Jiang Tan, Jia Yi Shen, Zhi En Chan, Wilson Chan, Tushar Gosavi, Shih Hui Lim

**Affiliations:** 1 Department of Neurology, National Neuroscience Institute (Singapore General Hospital Campus), 20 College Road, Singapore, 169856 Singapore; 2 Department of Neurology, National Neuroscience Institute (Tan Tock Seng Hospital Campus), Singapore, 308433 Singapore; 3 Duke-NUS Medical School, Neuroscience Academic Clinical Programme, SingHealth Duke-NUS Academic Medical Centre, Singapore, 169857 Singapore

**Keywords:** Febrile infection-related epilepsy syndrome, FIRES, Adult, Mortality, Survival

## Abstract

**Introduction:**

Febrile infection-related epilepsy syndrome (FIRES) is a subtype of new onset refractory status epilepticus (NORSE) that has, in recent years, been recognized to not only affect pediatric but adult patients too. While inpatient mortality in adult-onset FIRES has been suggested to be higher than in pediatric patients, little is known about the factors associated with survival, hampering research into developing prognostic and stratification models and also posing a challenge for clinical communications and decision making.

**Methods:**

We report a fatal case of FIRES in a 21-year-old lady and performed a review of literature involving adult-onset FIRES from 1 Aug 2022 to 17 Aug 2024, analyzing the data to identify factors associated with survival of the initial hospital stay.

**Results:**

Including our case, 49 patients of adult-onset FIRES were identified. Of the 47 patients who had MRI brain scan findings described at any time during the illness, 35 patients (74.5%) had abnormal findings in at least one scan, and the most common site of abnormality was the temporal lobe (59.6%). A total of 41 patients survived the initial hospital stay (83.7%). The presence of MRI temporal lobe abnormalities was associated with survival at discharge (*p* = 0.013), and the association remained significant on multivariate analysis with corticosteroid usage.

**Conclusion:**

In adult-onset FIRES, MRI temporal lobe abnormalities may be associated with survival beyond the acute phase. Our study findings suggest that the site of MRI abnormalities in FIRES may have value in the clinical stratification and prognostication of adult-onset FIRES.

**Supplementary Information:**

The online version contains supplementary material available at 10.1007/s10072-025-08728-0.

## Introduction

Febrile infection-related epilepsy syndrome (FIRES) is a subtype of new onset refractory status epilepticus (NORSE) characterized by a recent antecedent febrile illness [1–3]. In recent years, FIRES has been recognized to afflict patients of all ages, rather than predominantly pediatric patients, as was previously thought [2, 3].

In pediatric FIRES, mortality rate has been reported to be 11.7%, with longer periods of endotracheal intubation being associated with a poor outcome [4, 5]. Amongst adult-onset FIRES, acute phase mortality rate amongst in cryptogenic cases has been suggested to be higher at 26% [6]. Unlike in NORSE where some predictors of outcome have been identified, little is known about the factors associated with survival of adult-onset FIRES, much less its predictors [7, 8]. This knowledge gap leads to difficulty in risk-stratifying FIRES patients, which in turn adversely impacts clinicians’ ability to effectively counsel next-of-kin and set appropriate treatment goals and expectations.

We present a fatal case of FIRES in an adult, as well as the results of our review of literature surrounding cases of adult-onset FIRES, with the objective of identifying factors associated with survival of the initial hospitalization.

## Case presentation

One week after a febrile illness with cough and rhinorrhoea, a 21-year-old lady, with no significant medical, family, or recent vaccination history was sent by ambulance to the Emergency Department following a generalized tonic–clonic (GTC) seizure. At presentation, she was drowsy (Glasgow coma scale score of E2 V2 M5). She was unable to respond meaningfully to verbal or visual stimuli, but intermittently showed spontaneous purposeful movements with no obvious lateralized weakness. She had multiple GTC seizures with no recovery of consciousness in between, despite the administration of repeated doses of intravenous (IV) lorazepam and initiation of levetiracetam. Endotracheal intubation was thus performed and a continuous IV midazolam infusion was initiated. Lumbar puncture performed within the first 24 h of seizure onset showed normal cerebrospinal fluid (CSF) cell counts (red blood cells 0/mm^3^, white blood cells 3/mm^3^) and protein concentration (0.35 g/L). Despite her treatment, she continued to have multiple focal seizures, manifesting as clonic jerking or twitching of the right or left face, upper limb, and lower limb, which were terminated with boluses of IV diazepam. The patient was thereafter transferred to our centre for continuous electroencephalography (EEG) monitoring.

**Fig. 1:**
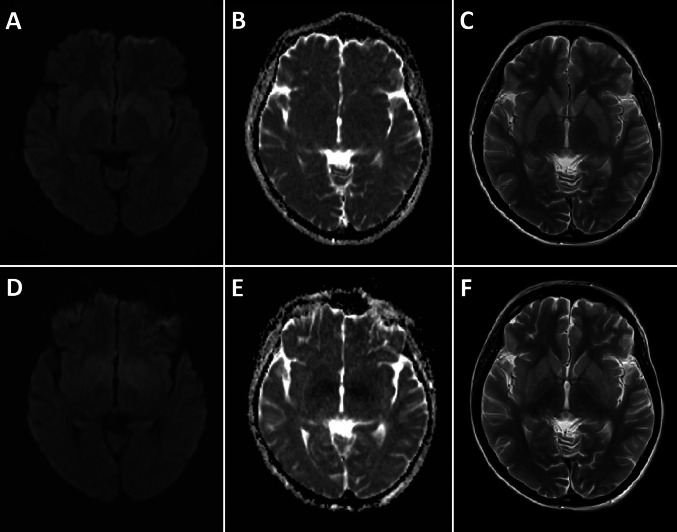
Magnetic resonance imaging (MRI) scans of the brain on day 3; **A**: Diffusion weighted imaging (DWI), **B:** apparent diffusion coefficient (ADC), and **C**: T2 weighted images; all normal. MRI brain scans on day 19; **D:** DWI, **E:** ADC, and** F**: T2 weighted images, showing poorly demarcated areas of restricted diffusion with corresponding ADC hypointensity and T2 hyperintensity in the bilateral basal ganglia and sub-insular regions (including the claustra)

A magnetic resonance imaging (MRI) scan of the brain performed on day 3 of seizure onset was normal (Fig. 1). EEG showed seizures of both generalised and focal onset, originating from both the right and left hemispheres. Apart from a borderline anti-nuclear antibody (ANA) titre of 1:160 (homogeneous, detected Hep-2 cells) and an indeterminate anti-double stranded DNA (anti-dsDNA) titre of 29.33 IU (reference range for positive > 30 IU, performed using enzyme immunoassay), blood and CSF panels for autoimmune encephalitis, paraneoplastic, and other serological antibodies were unyielding (see Table S1 of supplementary information). In lieu of the borderline ANA and anti-dsDNA results, further history was taken from family members, and clinical examination performed for signs of systemic lupus erythematosus (SLE), but these were unyielding. No history or signs of arthritis, arthralgia, rashes, oral ulcers, pleuritis, or pericarditis were found. A review of her previous blood test results did not show any history of hematologic abnormalities or renal impairment.

The patient initially received broad spectrum central nervous system (CNS) anti-microbial coverage, including IV ceftriaxone, vancomycin, and acyclovir. These were discontinued when the blood and CSF microbiological tests did not identify any active infection. Positive polymerase chain reaction (PCR) and aerobic culture tests for streptococcus pneumoniae in the patient’s endotracheal tube aspirate were thought to represent commensal rather than pathologic micro-organisms, given that the patient’s chest-X-ray and inflammatory markers (c-reactive protein and procalcitonin) were normal (see Table S1 of supplementary information).

Having established a diagnosis of cryptogenic FIRES, a five-day course of IV methylprednisolone followed by slow tapering of oral prednisolone was administered. Five cycles of plasma exchange therapy were completed across 11 days. Burst-suppression was achieved on EEG with the use of IV propofol and thiopental, in addition to multiple anti-seizure medications (ASMs), which included levetiracetam, sodium valproate, lacosamide, and phenytoin.

Treatment-related complications include hypotension requiring dual inotropic support, and intestinal hypomotility as evidenced by dilated bowel loops without obstruction on computed tomography (CT) scan of the abdomen. There were also medication-related hypernatremia, rhabdomyolysis, and deranged liver enzymes, resulting in discontinuation of lacosamide and phenytoin. Otherwise, there were no signs of other medication-related complications such as propofol infusion syndrome (PRIS); During the period of propofol infusion, there was a downward trend of creatine kinase, and no signs of metabolic acidosis or kidney injury on daily blood tests.

As the patient remained comatose without clinical seizures, and EEG continued to showed burst-suppression, IV propofol and thiopental were weaned off. Between days 11 and 17, the patient had fixed and dilated pupils, likely due to the long half-life of the recently discontinued IV thiopental. On day 18, despite the ongoing use of levetiracetam and sodium valproate, clinical and EEG seizures recurred. This prompted the introduction of IV ketamine, which achieved a state of EEG suppression. However, as clinical seizures recurred on attempted weaning of ketamine, multiple oral ASMs were rapidly added over the next two days (phenobarbital, topiramate, lamotrigine, and perampanel). Ketogenic diet was not attempted due to concerns surrounding the active issues of hypotension, intestinal pseudo-obstruction, and deranged liver enzymes. On day 19, a repeat MRI brain scan showed T2 hyperintense signal abnormalities involving the bilateral basal ganglia and sub-insular regions (including the claustra), with poorly demarcated and diffuse areas of restricted diffusion and corresponding apparent diffusion coefficient (ADC) hypointensity (Fig. 1).

Although serial electrocardiogram (ECG) recordings did not show any significant abnormalities, with corrected QT interval fluctuating between 355 and 426 ms, the patient suffered a sudden asystolic cardiac arrest on day 21 and demised on the same day.

## Methods

Noting that no Medical Subject Heading (MeSH) terms for FIRES or NORSE are currently indexed, two reviewers working independently performed searches on PubMed using the terms “febrile infection-related epilepsy syndrome” and “new onset refractory status epilepticus”. All reviewers were registered as practising Neurologists with the Singapore Medical Council, and were International League against Epilepsy Asian Epilepsy Academy (ILAE ASEPA) EEG certified interpreters, at the time of the literature review.

The inclusion criteria were (1) the paper was published in English after 1 Aug 2022, (2) the study design was a case report, case series, or cohort study with descriptions of unique case(s), (3) the patient(s) described were adults of age more than or equal to 18-years-old, and (4) the case description was consistent with a diagnosis of FIRES. The date of 1 Aug 2022 was chosen because the last International Consensus statements and summary on FIRES were published in Aug 2022 [2, 3]. The definition of “adult” was taken to be at least 18 years-old because previous studies on pediatric FIRES and its mortality have mostly included patients under 18 years of age [4]. Studies which described both pediatric and adult-onset patients of FIRES were included, but only data for the adult patients were collected. We defined FIRES as a subcategory of NORSE with a prior febrile infection starting between two weeks and 24 h prior to the onset of RSE, with no requirement for the fever to persist at the onset of seizures [1–3]. NORSE was defined as de novo onset of refractory status epilepticus in a patient without active epilepsy or other pre-existing relevant neurological disorder, and without an identifiable acute or active structural, toxic or metabolic cause [1–3].

The exclusion criteria were (1) studies in which the age of each reported subject could not be ascertained, (2) studies which had no description of survival outcome for each subject, and (3) cases which had descriptions that were insufficiently precise, such that confirmation of FIRES using the above definition was precluded.

The demographical data, details of antecedent vaccines or illnesses, presence of headache at any time, EEG findings, timing and findings of initial MRI brain scan, sites of MRI brain abnormalities (occurring at any time during the admission), serological and CSF test results, etiology of FIRES, treatment administered, duration of hospitalization, and eventual outcomes were manually collected on spreadsheets. Initial MRI brain was defined as the first MRI brain scan done after seizure onset. The duration of hospitalization was measured from the day of presentation to the day of death or discharge. Discharge was defined as discharge from the index inpatient hospital stay or discharge to a rehabilitation unit. The primary outcome was survival at the time of discharge. Amongst survivors, last-reported modified Rankin scale (mRS) score and the presence of drug-resistant epilepsy (DRE) at last follow-up were also recorded, if they were described. DRE was defined as failure of two tolerated, appropriately chosen ASM to achieve at least 12 months of seizure freedom [9]. For the purpose of defining DRE in our study, the alternative definition of seizure freedom based on pre- and post-intervention inter-seizure intervals was not used, because FIRES patients do not have pre-existing epilepsy [3, 9]. For EEG and MRI brain data, both text descriptions and published images were reviewed. Disagreements between the reviewers regarding inclusion, exclusion, or data collected were resolved by a third reviewer.

Data were analyzed against an outcome of death before discharge using SPSS (version 26.0, Chicago, IL, USA). The Mann–Whitney U test was used for ordinal variables, while the Pearson Chi-square or Fisher’s exact tests were used for categorical variables. Multivariate analysis was selectively carried out using multinomial logistic regression, based on any suspected confounding effect between the variables analyzed; For this purpose, ordinal variables were categorized by their relationship to the mean value.

## Results

**Fig. 2:**
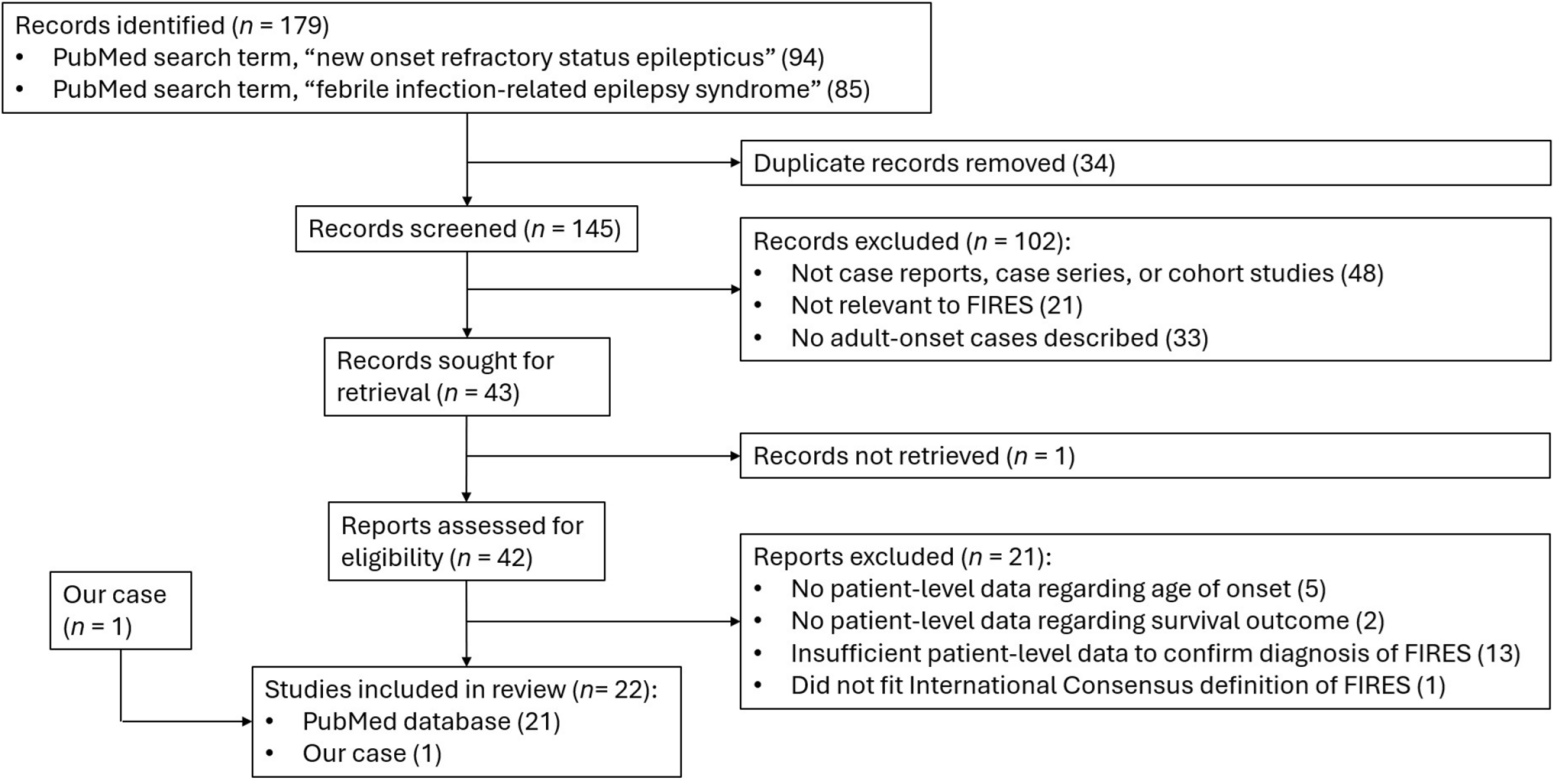
PRISMA flowchart detailing each stage of our literature search

The literature search concluded on the 27th of August 2024, with 179 search results. After removing 34 duplicates, 145 unique articles were identified. After application of our inclusion and exclusion criteria, and the removal of one article which could not be retrieved, 21 articles were included. Figure 2 represents a PRISMA flowchart providing further details about our literature search.

The 21 articles comprised 15 case reports, five cohort studies, and one case series. They described a total of 48 patients of adult-onset FIRES, and our patient was added as the 49th case [10–30].

### Clinical features

The characteristics of all identified patients are described in Table 1, while the clinico-radiologic characteristics and mortality outcomes of each case are detailed in Table S2 of the supplementary information. The median age was 25 years-old (range 18 to 65), and 29 patients (59.2%) were female. A minority (8/49, 16.3%) had a specific antecedent vaccine or illness described, which were one of the following: Severe acute respiratory syndrome coronavirus 2 (SARS CoV2) vaccines, COVID-19 infection, or mycoplasma infection. The interval between the antecedent event and first presentation was only described in three of these cases, all of them being SARS CoV2 vaccines, and with the interval ranging from 8 days to 2 months. The number of days between fever onset and seizure onset was reported in 37 patients, and the median was five days (range 2 to 10 days).

**Table 1:** Characteristics of the patients of adult-onset febrile infection-related epilepsy syndrome (FIRES) identified. CSF, cerebrospinal fluid; IVIG, intravenous immunoglobulin; MRI, magnetic resonance imaging; SARS CoV2, severe acute respiratory syndrome coronavirus 2; *SD*, standard deviation; WBC, white blood cell

Characteristics	*N* (data available)		*n* or median (% or range)
Age at presentation (years)	49		25 (18–65)
Sex	49	Male	20 (40.8%)
		Female	29 (59.2%)
Specified antecedent event	8	SARS CoV2 vaccine	6 (75.0%)
		COVID-19 infection	1 (12.5%)
		Mycoplasma infection	1 (12.5%)
Time from fever onset to seizure onset (days)	37		5 (2–10)
Headache at any time	49		12 (24.5%)
EEG bilateral or multifocal seizures	32		23 (71.9%)
Initial MRI brain scan was normal	28		18 (64.3%)
Initial MRI brain scan within 3 days of seizure onset was normal	16		10 (62.5%)
MRI brain showed claustrum sign at any time	45		16 (35.6%)
Sites of MRI brain abnormalities	47	Frontal lobe	11 (23.4%)
		Temporal lobe	28 (59.6%)
		Parietal lobe	7 (14.9%)
		Occipital lobe	6 (12.8%)
		Thalamus	3 (6.4%)
		Corpus callosum	3 (6.4%)
		Basal ganglia	3 (6.4%)
		Cerebellum	2 (4.3%)
		Two or more sites of abnormalities	17 (36.2%)
CSF WBC more than 5 per mm^3^	43		20 (46.5%)
CSF protein more than 0.4 g/L	33		15 (45.5%)
Etiology of FIRES	49	Known etiology	3 (6.1%)
		Cryptogenic	46 (93.9%)
Anti-seizure treatment administered	31	Levetiracetam	30 (96.8%)
		Phenytoin	14 (45.2%)
		Valproate	20 (64.5%)
		Carbamazepine	9 (29.0%)
		Oxcarbazepine	3 (9.7%)
		Topiramate	19 (61.3%)
		Lamotrigine	5 (16.1%)
		Phenobarbital	22 (71.0%)
		Pentobarbital	2 (6.5%)
		Lacosamide	15 (48.4%)
		Perampanel	14 (45.2%)
		Zonisamide	2 (6.5%)
		Rufinamide	2 (6.5%)
		Vigabatrin	1 (3.2%)
		Pregabalin	2 (6.5%)
		Gabapentin	3 (9.7%)
		Cannabidiol	1 (3.2%)
		Propofol	18 (58.1%)
		Ketamine	10 (32.3%)
		Dexmedetomidine	1 (3.2%)
		Thiopental	8 (25.8%)
		Ketogenic diet	7 (22.3%)
Immunotherapy administered	49	Corticosteroids	43 (87.8%)
		IVIG	34 (69.4%)
		Plasma exchange	17 (34.7%)
		Rituximab	10 (20.4%)
		Cyclophosphamide	3 (6.1%)
		Tocilizumab	5 (10.2%)
Duration of hospitalization ≥ 60 days	19		13 (68.4%)
Survival of initial hospitalization	49		41 (83.7%)
mRS 0–3 at last follow-up (amongst survivors)	32		17 (53.1%)
Drug-resistant epilepsy at last follow-up (amongst survivors)	30		23 (76.7%)

### Investigation findings

Descriptions of EEG findings were found in 34 patients, out of which there were 17 patients (50%) with bilateral EEG seizures and nine patients (26.5%) with either unilateral EEG seizures or no EEG seizures. A further six patients (17.7%) had multifocal EEG seizures without further specification of laterality, and two patients (5.9%) had less precise EEG descriptions that were not classifiable into any of the aforementioned categories. Hence, 23 out of 32 classifiable patients (71.9%) had either bilateral or multifocal EEG seizures.

Initial MRI brain scans were described in 28 patients, of whom 18 patients (64.3%) had a normal initial scan. Amongst these 18 patients whose initial MRI brain scans were normal, seven patients (38.9%) had subsequent MRI brain scans that turned abnormal. The time between seizure onset and the initial MRI brain scan was described in 19 patients, with the median being 1 day (*SD* = 4.0, range 1 to 14 days). There were 16 patients who had their initial MRI brain scan performed within three days of seizure onset, and 10 of these (62.5%) were normal.

Amongst the 47 patients who had MRI brain scan findings described at any time during the illness (including both initial and follow-up scans), 35 patients (74.5%) had abnormal findings in at least one scan and 17 patients (36.2%) had two or more sites of abnormalities. The most common site of abnormality was the temporal lobe (59.6%). Two patients were described to have insular abnormalities, but we were unable to determine if these also involved the claustrum. Amongst the remaining 45 patients, 16 patients (35.6%) had claustrum abnormalities at some point of time during the disease course.

Out of 43 patients which had reported CSF cell counts, there was CSF pleocytosis in 20 patients (46.5%). There were 31 patients with a precisely reported CSF WBC count (median 7 per mm^3^, range 0 to 200), and 19 of these (61.3%) had a CSF WBC of < 10 per mm^3^. CSF protein was elevated in 15 of 33 patients (45.5%) who had CSF protein data reported. There were 29 patients with a precisely reported CSF protein concentration (median 0.4 g/L, range 0.15 to 1.24), and a majority of 25 (86.2%) had CSF protein concentrations of < 0.6 g/L. Only five patients had CSF differential count described, and all were lymphocytic.

Only three patients (6.1%) had a known etiology (detailed in Table S2 of supplementary information). These etiologies include two cases of anti-NMDAR encephalitis (one with concomitant Japanese encephalitis), and one case of MT-TF gene mutation. The etiology could not be definitively identified in the remaining patients, which were thus categorized as cryptogenic FIRES, including one case with underlying TNFRSF13B mutation-related common variable immunodeficiency.

### Treatment administered

ASM data were available in 31 patients, and the median number of different ASMs used was 5 (range 1 to 8). The most used ASM was levetiracetam (96.8%). The median number of anesthetic agents used was 3 (range 0 to 3), and the most used anesthetic agent was propofol (58.1%). Ketogenic diet was instituted in 22.3%.

The most used form of immunotherapy was corticosteroids (87.8%), while IVIG (69.4%) and plasma exchange (34.7%) were the next most common.

### Outcomes

A precise duration of hospitalization was only available in nine patients, and the average was 97 days (standard deviation, *SD* = 68.5, range 21 to 230). However, 19 patients had descriptions that were at least categorizable, based on the descriptions and contextual information provided, into whether the duration of hospitalization was < 60 days, or ≥ 60 days. Amongst these 19 patients, 13 patients (68.4%) had a duration of hospitalization that was ≥ 60 days.

A total of 41 patients (83.7%) survived the initial hospitalization, out of whom only 15 patients had categorizable durations of hospitalization. Twelve out of these 15 survivors (80%) had hospital stays longer than 60 days.

Amongst the survivors, 32 patients had mRS reported at the last follow-up, and 17 of these survivors (53.1%) had an mRS of 0 to 3. The duration of the last follow-up was reported in 22 patients, and the median duration was 52 weeks (range 6 to 468 weeks). One patient who survived the initial admission, having been discharged with an mRS of 4, was dead at 20-week follow-up; This was the only case of death after discharge.

Amongst the survivors, 30 patients had seizure control reported at the last follow-up, and 23 of these survivors (76.7%) had DRE. The duration of the last follow-up was reported in 27 patients, and the median duration was 48 weeks (range 4 to 468 weeks).

For the eight patients who did not survive the initial hospitalization, the definitive mechanism of death was uncertain (including our patient), though three of them were described to have suffered from combinations of sepsis, liver failure, heart failure, and anemia. Our patient had suffered a cardiac arrest, but the exact cause of her cardiac arrest was uncertain.

### Analysis results

Selected variables and the *p*-values from their analyses against an outcome of survival at discharge are shown in Table 2. The results of analyses of the other variables are available in Table S3 of the supplementary information.

**Table 2:** Selected results from analyses of variables against an outcome of death before discharge, amongst adult patients with febrile infection-related epilepsy syndrome (FIRES) of any etiology. MRI, magnetic resonance imaging; OR, odds ratio

Variables	*n*	OR	*p*-value
MRI temporal lobe abnormalities at any time	47	12.5	0.013
Corticosteroids used	49	7.6	0.047
Multivariate analysis	*n*	OR	*p*-value
MRI temporal lobe abnormalities at any time	47	16.7	0.025
Corticosteroids used	47	8.9	0.104

Temporal lobe abnormalities on MRI brain scan and the administration of corticosteroids were associated with survival at discharge (odds ratio 12.5 and 7.6, *p* = 0.013 and 0.047 respectively). Table 3 shows the number of patients categorized by MRI temporal lobe abnormalities, corticosteroid use, and survival.

**Table 3:** Adult-onset febrile infection-related epilepsy syndrome (FIRES) patients categorized by the presence of temporal lobe abnormalities on magnetic resonance imaging of the brain, corticosteroid use, and survival of the initial hospitalization. MRI, magnetic resonance imaging

MRI temporal lobe abnormalities at any time (*n* = 47)			
		Survived initial hospitalization	Death before discharge
MRI temporal lobe abnormalities	Present	27	1
	Absent	13	6
Corticosteroids used (*n* = 49)			
		Survived initial hospitalization	Death before discharge
Corticosteroids	Used	38	5
	Not used	3	3

On a suspicion that the variables of MRI temporal lobe abnormalities and use of corticosteroids were mutual confounders, a multivariate analysis was performed using these variables against an outcome of survival at discharge. On this multivariate analysis, MRI temporal lobe abnormalities retained a statistically significant association with survival at discharge (*p* = 0.025), while the use of corticosteroids became non-significant as an association (*p* = 0.104).

## Discussion

By definition, NORSE and FIRES are not due to structural, toxic, or metabolic causes [1]. Despite an extensive evaluation, most cases remain cryptogenic, and our study provided further support for this observation [31]. Although a definitive etiology may remain elusive in cryptogenic cases, both innate and adaptive immune responses have been implicated in its pathophysiology [2, 32]. It is therefore unsurprising that some cases of NORSE or FIRES, such as our case, can fulfil proposed diagnostic criteria for possible autoimmune encephalitis [33].

Although corticosteroid usage was significantly associated with survival in our initial univariate analysis, we were cautious about its generalizability to all FIRES patients, given that the evidence for corticosteroid use remains weak in FIRES [34]. Noting that MRI temporal lobe abnormalities were also associated with survival in our analysis, we postulated that the higher survival amongst FIRES patients given corticosteroids was driven by unidentified steroid-responsive autoimmune encephalitis with a predilection for the temporal lobes. Cell-surface autoantibody-related encephalitis represents an example of a type of encephalitis that has a predilection for the temporal lobes and is also more likely to respond to immunomodulatory therapy [35]. This was the rationale behind the selection of variables for our second multivariate analysis, which seemed to support our suspicion, since corticosteroid usage lost its statistical significance as an associated factor while MRI temporal lobe abnormalities retained its association.

Our study did not involve a direct comparison between pediatric and adult-onset FIRES, but there were some observations that may be worth investigating further. Compared to a previous study of pediatric FIRES, our study potentially suggested that adult-onset FIRES had a higher proportion of corticosteroid receivers (87.8% vs 63.0%), less DRE amongst survivors (76.7% vs 93%), and a higher acute phase mortality (16.3% vs 11.7%) [4].

The higher proportion of adult-onset FIRES receiving corticosteroids may be because NORSE is thought to be more commonly autoimmune-driven amongst adults compared to children, and corticosteroids represent a first-line form of immunotherapy in NORSE [2, 36]. The lower incidence of DRE amongst survivors of adult-onset FIRES may similarly be related to a difference between the underlying pathogenesis of adult-onset and pediatric FIRES. This hypothesis is supported by studies showing that the prevalence of persistent epilepsy and refractory epilepsy after encephalitis varies by etiology in both children and adults, and that autoimmune forms of encephalitis may have a lower risk of subsequent refractory epilepsy [37, 38].

The higher acute phase mortality in adult-onset FIRES compared to pediatric FIRES is consistent with the findings of previous studies, but compared to a recent adult-predominant study of FIRES by Jimenez et al., our study had a lower inpatient mortality (16.3% vs 26%) [6]. The 34 FIRES patients from Jimenez et al.’s study were excluded from our review because patient-level data was unavailable to us. Although it included a few pediatric FIRES patients (4/34, 12%), Jimenez et al.’s cohort had an otherwise similar age profile to the patients in our study (median age 28 vs 25). We were unable to perform any association analysis without patient-level data, but we noted that the lower proportion of temporal lobe abnormalities in their study compared to our study (38% vs 59.6%) and the higher inpatient mortality rate in their study appear consistent with our finding that temporal lobe abnormalities could be associated with survival.

Our study has several limitations which mean that the results must be interpreted with caution. First, our sample size is relatively small, hence limiting the generalizability of our findings. The small sample size also meant that a broader multivariate analysis to identify more confounding factors would greatly reduce the statistical power of the data, preventing any meaningful interpretation. We sought to mitigate this problem through a carefully considered approach to selecting potential confounders.

We analyzed our data by site of MRI abnormality, but we were unable to obtain more detailed information that would have enabled an analysis based on specific MRI sequence characteristics of the abnormalities reported, due to a lack of a standardized data reporting protocol amongst the studies we included. This would have been useful information, given that the type of a given MRI abnormality is likely to impact its association with acute phase mortality. For instance, the presence of peri-ictal MRI abnormalities, characterized most often by restricted diffusion or T2 hyperintensity of the cerebral cortex with variable findings on ADC sequence, is associated with higher mortality in status epilepticus, perhaps reflecting seizure-induced mitochondrial dysfunction and neuronal necrosis [39]. However, our analysis by site of MRI abnormalities remain potentially useful information, given that there are no existing prognostic models for adult-onset FIRES, and current prognostic models for status epilepticus in general (such as the STESS and EMSE scores) do not take into account the sites of MRI abnormalities [40, 41].

The limitation of not having a standardized data reporting protocol extended beyond MRI data to other variables, such as the duration of hospitalization, EEG findings, CSF findings, treatment details, and outcomes. For example, some reports described the duration of hospitalization in weeks, some in months, and others described the duration of intensive care unit (ICU) stay but not hospitalization. Wide-ranging heterogeneity in the scope and depth of each report meant that patients with vague descriptions had to be excluded from the analyses of certain variables. We sought to mitigate this problem by categorizing variables whenever we could, so that less precisely described patients could still be included if the information sufficed for categorization. However, there was a limit to the extent to which categorization was feasible. For instance, narrative cognitive and epilepsy refractoriness outcome data were collected, but there was no feasible way to categorize the highly heterogeneous (and often incomplete) descriptions. Another example of incomplete data was the lack of precise information concerning the dosages, durations, and sequences of immunotherapy and anesthetic agents used, hence preventing us from better understanding the context, therapeutic roles, and prognostic value of the treatment used in each case.

For the patients who demised, the cause of death would have been of interest to our study. However, we found that the specific cause of death was either not clearly specified or uncertain for the mortality cases we identified. In our patient, the final event leading to death was cardiac arrest, but the exact etiology remained unclear. Although there was no obvious QT prolongation in the ECGs recorded, it is possible that the additive effect of sodium channel blockers used for seizure control may have contributed to cardiac arrhythmia. An alternative explanation is that insular region abnormalities may have led to cardiac dysregulation [42]. While FIRES patients typically have significant complications from both the disease and treatment during hospitalization, the ultimate mechanism of death can sometimes be speculative, and this is an area that may be worth further attention and research [4].

The retrospective design of our study and its dependence on published cases made it potentially prone to biases. For example, amongst the patients which had an antecedent vaccine or illness described, a majority were either related to SARS-CoV2 vaccine or COVID-19 infection, an observation that may be related to publication bias driven by the relative novelty of COVID-19. We also wonder if there might be a publication bias favoring surviving cases, which may explain the relatively low mortality in our study, potentially affecting our finding of MRI temporal lobe abnormalities being associated with survival. Similarly, there may also be a publication bias favoring cryptogenic cases, since cases with known etiologies that have been well studied may be less likely to be published (such as anti-NMDAR encephalitis).

Nonetheless, we hope that our study may serve as a springboard for future research into prognostic stratification of adult-onset FIRES and provide some basis for clinicians to counsel next-of-kin and make informed clinical decisions when faced with this often-devastating syndrome. Our hypothesis that steroid-responsive etiologies may drive the association between MRI temporal lobe abnormalities and survival in FIRES should also be explored in future studies. Finally, a comparative analysis between adult-onset and pediatric FIRES may provide additional insights into potential differences in pathophysiology, treatment response, and outcomes between the two populations.

## Conclusions

We added a fatal case of adult-onset FIRES to the literature and described the results of our review of this syndrome. In adult-onset FIRES, the presence of MRI temporal lobe abnormalities may be associated with survival at discharge, potentially because temporal lobe abnormalities are a marker for an underlying corticosteroid-responsive immune-mediated etiology. In addition, our study corroborated a higher inpatient mortality rate amongst adult-onset FIRES compared to pediatric FIRES, though the exact mortality rate for the former remains uncertain.

## Supplementary Information

Below is the link to the electronic supplementary material.

Supplementary file1 (DOCX 18 KB)

Supplementary file2 (DOCX 23 KB)

Supplementary file3 (DOCX 18 KB)

## Data Availability

The data used in this study has either been availed through the manuscript and supplementary information, or is available from the cited literature.
