# Supplementary material for: Febrile infection-related epilepsy syndrome (FIRES) in adults: a case report and review of factors associated with survival
Source: Neurol Sci. 2026 Jan 12;47(1):147. doi: 10.1007/s10072-025-08728-0 (PMC12795905; doi:10.1007/s10072-025-08728-0)
Supplement: Supplementary file 1 — Supplementary file1 (DOCX 18 KB) [file 10072_2025_8728_MOESM1_ESM.docx]

**Table S1**. Summary of pertinent investigation results for our patient.

| **Blood hematological and biochemical tests** | **Result** |
| --- | --- |
| Hemoglobin | 13.0g/dL |
| White blood cell count | 10.2 x 10^9^/L |
| Platelets | 145 x 10^9^/L |
| Urea, creatinine and electrolytes | Normal |
| Liver enzymes | Normal |
| C-reactive protein and procalcitonin | Normal |
|  |  |
| **Blood serological tests** | **Result** |
| Anti-nuclear antibody (ANA) | 160 (homogeneous) |
| Anti-double stranded DNA antibody | 29.33 IU  (indeterminate) |
| Anti-neutrophil cytoplasmic antibody (ANCA) | Negative |
| Anti-ENA profile, comprising antibodies against Smith, ribonucleoprotein, Ro, La, Scl 70, and Jo-1 | Negative |
| Autoimmune encephalitis panel, comprising antibodies against NMDAR, CASPR2, AMPAR, LGI1, DPPX, and GABA_B_R | Negative |
| Onconeural panel, comprising antibodies against amphiphysin, CV2, Ta, Ri, Yo, Hu, Recoverin, SOX1, Titin, Zic4, GAD65, and Tr | Negative |
|  |  |
| **CSF tests** | **Result** |
| White cell count | 3 /mm^3^ |
| Red blood cell count | Not detected |
| Protein | 0.35 g/L |
| CSF microbiological tests, including gram stain, culture, FilmArray meningitis PCR panel, VDRL, syphilis IgG, AFB smear, AFB culture, TB DNA PCR, fungal culture, cryptococcus antigen, HSV isolation, measles isolation, mumps isolation, enterovirus isolation, HSV antibody, measles antibody, mumps antibody | Negative |
| CSF autoimmune encephalitis panel, comprising antibodies against NMDAR, CASPR2, AMPAR, LGI1, DPPX, and GABA_B_R | Negative |
|  |  |
| **Blood microbiological tests** | **Result** |
| Blood cultures (aerobic and anaerobic) | Negative |
| HIV antigen/antibody immunoassay | Negative |
| Tuberculosis IGRA | Negative |
| VDRL | Negative |
|  |  |
| **Other microbiological tests** | **Result** |
| Urine culture | Negative |
| Nose and throat swab Biofire Filmarray Respiratory Pathogens 2.1 PCR panel (including SARS-CoV-2) | Negative |
| Endotracheal tube aspirate Seegene Anyplex II RB5 Pneumonia PCR panel | Negative |
| Endotracheal tube aspirate Biofibre Filmarray Pneumonia PCR panel | *Streptococcus pneumoniae* (high DNA load) |
| Endotracheal tube aspirate respiratory culture | Streptococcus pneumoniae, 10,000 to 100,000 cfu/mL |
