# Supplementary material for: Febrile infection-related epilepsy syndrome (FIRES) in adults: a case report and review of factors associated with survival
Source: Neurol Sci. 2026 Jan 12;47(1):147. doi: 10.1007/s10072-025-08728-0 (PMC12795905; doi:10.1007/s10072-025-08728-0)
Supplement: Supplementary file 2 — Supplementary file2 (DOCX 23 KB) [file 10072_2025_8728_MOESM2_ESM.docx]

**Table S2**. Clinicoradiologic characteristics and mortality outcomes of adult FIRES patients collated from literature review and our case report. CVID, common variable immunodeficiency; FIRES, febrile infection-related epilepsy syndrome; JEV, Japanese encephalitis virus; MRI, magnetic resonance imaging; NMDAR, N-methyl-D-aspartate receptor.

| Patient | Ref | Age at FIRES onset (years) | Sex | Aetiology | Sites of MRI brain abnormalities (at any time) | Survival at discharge |
| --- | --- | --- | --- | --- | --- | --- |
| 1 | Girardin ML et al.[8] | 22 | Male | Cryptogenic | Claustrum, temporal lobe corpus callosum | Yes |
| 2 | Watanabe T et al.[9] | 33 | Female | Cryptogenic | Frontal and temporal lobes | Yes |
| 3 | Werner J et al.[10] | 35 | Female | Cryptogenic | Claustrum, temporal lobe | Yes |
| 4 | Indelicato E et al.[11] | 29 | Female | MT-TF gene mutation (mitochondrial disorder) | Frontal, temporal, parietal, occipital lobes, thalamus, basal ganglia, cerebellum | Yes |
| 5 | Haanpää A et al.[12] | 25 | Male | Cryptogenic | Nil | Yes |
| 6 | Haanpää A et al.[12] | 19 | Male | Cryptogenic | Nil | Yes |
| 7 | Haanpää A et al.[12] | 20 | Female | Cryptogenic | MRI brain data unavailable | No |
| 8 | Haanpää A et al.[12] | 18 | Male | Cryptogenic | Nil | No |
| 9 | Haanpää A et al.[12] | 28 | Female | Cryptogenic | Nil | Yes |
| 10 | Haanpää A et al.[12] | 21 | Female | Cryptogenic | Nil | Yes |
| 11 | Alemdar M et al.[13] | 31 | Female | NMDAR encephalitis | Nil | Yes |
| 12 | Osbourn SR et al.[14] | 37 | Female | JEV and anti-NMDAR encephalitis | Temporal lobe, thalamus | Yes |
| 13 | Li H et al.[15] | 20 | Male | Cryptogenic | Frontal, temporal, and parietal lobes, cerebellum | Yes |
| 14 | Li H et al.[15] | 65 | Female | Cryptogenic | Temporal lobe, basal ganglia | No |
| 15 | Li H et al.[15] | 22 | Male | Cryptogenic | Nil | Yes |
| 16 | Kwack DW et al.[16] | 24 | Female | Cryptogenic | Nil | Yes |
| 17 | Nakamura Y et al.[17] | 35 | Female | Cryptogenic | Claustrum, frontal, temporal, and parietal lobes | Yes |
| 18 | Yang JH et al.[18] | 21 | Female | Cryptogenic, with underlying TNFRSF13B mutation-related CVID | Temporal lobe | Yes |
| 19 | Oliger A et al.[19] | 33 | Male | Cryptogenic | Temporal lobe | Yes |
| 20 | Oliger A et al.[19] | 26 | Male | Cryptogenic | Temporal lobe | Yes |
| 21 | deCampo D et al.[20] | 18 | Female | Cryptogenic | MRI brain data unavailable | Yes |
| 22 | Sugata M et al.[21] | 20 | Female | Cryptogenic | Frontal, parietal, and occipital lobes | Yes |
| 23 | Mehboob S et al.[22] | 54 | Male | Cryptogenic | Frontal and temporal lobes | Yes |
| 24 | Espino PH et al.[23] | 37 | Female | Cryptogenic | Claustrum and temporal lobe | Yes |
| 25 | Shi X et al.[24] | 35 | Male | Cryptogenic | Nil | No |
| 26 | Shi X et al.[24] | 20 | Female | Cryptogenic | Frontal and temporal lobes | Yes |
| 27 | Shi X et al.[24] | 24 | Female | Cryptogenic | Nil | No |
| 28 | Shi X et al.[24] | 28 | Male | Cryptogenic | Nil | No |
| 29 | Shi X et al.[24] | 21 | Female | Cryptogenic | Frontal, parietal, and occipital lobes, corpus callosum | Yes |
| 30 | Shi X et al.[24] | 23 | Male | Cryptogenic | Temporal lobe | Yes |
| 31 | Shi X et al.[24] | 22 | Female | Cryptogenic | Nil | No |
| 32 | Shi X et al.[24] | 27 | Female | Cryptogenic | Temporal lobe, corpus callosum | Yes |
| 33 | Shi X et al.[24] | 30 | Male | Cryptogenic | Temporal and occipital lobes | Yes |
| 34 | Shi X et al.[24] | 30 | Female | Cryptogenic | Temporal lobe | Yes |
| 35 | Bai L et al.[25] | 20 | Female | Cryptogenic | Claustrum and temporal lobe | Yes |
| 36 | Bai L et al.[25] | 21 | Male | Cryptogenic | Claustrum | Yes |
| 37 | Bai L et al.[25] | 26 | Male | Cryptogenic | Claustrum, temporal and occipital lobes | Yes |
| 38 | Bai L et al.[25] | 23 | Female | Cryptogenic | Claustrum and temporal lobe | Yes |
| 39 | Bai L et al.[25] | 32 | Male | Cryptogenic | Claustrum and temporal lobe | Yes |
| 40 | Bai L et al.[25] | 42 | Female | Cryptogenic | Claustrum and temporal lobe | Yes |
| 41 | Bai L et al.[25] | 25 | Male | Cryptogenic | Claustrum, frontal and parietal lobes | Yes |
| 42 | Bai L et al.[25] | 64 | Female | Cryptogenic | Claustrum and temporal lobe | Yes |
| 43 | Bai L et al.[25] | 20 | Male | Cryptogenic | Claustrum | Yes |
| 44 | Bai L et al.[25] | 37 | Female | Cryptogenic | Claustrum and temporal lobe | Yes |
| 45 | Bai L et al.[25] | 42 | Female | Cryptogenic | Claustrum and temporal lobe | Yes |
| 46 | Goh Y et al.[26] | 18 | Male | Cryptogenic | Frontal, temporal, parietal, and occipital lobes | Yes |
| 47 | Aledo-Serrano A et al.[27] | 18 | Female | Cryptogenic | Temporal lobe | Yes |
| 48 | Safan AS et al.[28] | 30 | Male | Cryptogenic | Frontal lobe, thalamus | Yes |
| 49 | Our patient | 21 | Female | Cryptogenic | Claustrum, basal ganglia | No |
