# Supplementary material for: Febrile infection-related epilepsy syndrome (FIRES) in adults: a case report and review of factors associated with survival
Source: Neurol Sci. 2026 Jan 12;47(1):147. doi: 10.1007/s10072-025-08728-0 (PMC12795905; doi:10.1007/s10072-025-08728-0)
Supplement: Supplementary file 3 — Supplementary file3 (DOCX 18 KB) [file 10072_2025_8728_MOESM3_ESM.docx]

**Table S3**. Analyses of variables against an outcome of survival at discharge, amongst adult patients with febrile infection-related epilepsy syndrome (FIRES) of any etiology. Two-sided *p*-values are shown. Variables with statistically significant associations are in bold. CSF, cerebrospinal fluid; IVIG, intravenous immunoglobulin; MRI, magnetic resonance imaging; WBC, white blood cell.

| Variable | *n* | Test *p*-value |
| --- | --- | --- |
| Age at presentation | 49 | 0.664 |
| Sex | 49 | 1.000 |
| Time from fever onset to seizure onset | 37 | 0.056 |
| Headache at any time | 49 | 0.386 |
| EEG bilateral or multifocal seizures | 32 | 1.000 |
| Initial MRI brain normal | 28 | 0.364 |
| Initial MRI brain scan within 3 days of seizure onset was normal | 16 | 0.307 |
| MRI claustrum sign at any time | 45 | 0.393 |
| MRI frontal lobe abnormalities at any time | 47 | 0.175 |
| **MRI temporal lobe abnormalities at any time** | **47** | **0.013** |
| MRI parietal lobe abnormalities at any time | 47 | 0.573 |
| MRI occipital lobe abnormalities at any time | 47 | 0.571 |
| MRI thalamic abnormalities at any time | 47 | 1.000 |
| MRI corpus callosal abnormalities at any time | 47 | 1.000 |
| MRI basal ganglia abnormalities at any time | 47 | 0.054 |
| MRI cerebellar abnormalities at any time | 47 | 1.000 |
| Two or more sites of abnormalities | 47 | 0.396 |
| CSF WBC more than 5 per mm^3^ at any time | 43 | 0.704 |
| CSF protein more than 0.4 g/L | 33 | 1.000 |
| Cryptogenic etiology | 49 | 1.000 |
| Levetiracetam used (at any time) | 31 | 1.000 |
| Phenytoin used (at any time) | 31 | 0.185 |
| Valproate used (at any time) | 31 | 1.000 |
| Carbamazepine used (at any time) | 31 | 0.320 |
| Oxcarbamazepine used (at any time) | 31 | 1.000 |
| Topiramate used (at any time) | 31 | 0.363 |
| Lamotrigine used (at any time) | 31 | 1.000 |
| Phenobarbital used (at any time) | 31 | 0.642 |
| Pentobarbital used (at any time) | 31 | 1.000 |
| Lacosamide used (at any time) | 31 | 0.172 |
| Perampanel used (at any time) | 31 | 0.185 |
| Zonisamide used (at any time) | 31 | 1.000 |
| Rufinamide used (at any time) | 31 | 1.000 |
| Vigabatrin used (at any time) | 31 | 1.000 |
| Pregabalin used (at any time) | 31 | 1.000 |
| Gabapentin used (at any time) | 31 | 1.000 |
| Cannabidiol used (at any time) | 31 | 1.000 |
| Propofol used (at any time) | 31 | 0.359 |
| Ketamine used (at any time) | 31 | 0.358 |
| Dexmedetomidine used (at any time) | 31 | 1.000 |
| Thiopental or thiamylal used (at any time) | 31 | 1.000 |
| Ketogenic diet used (at any time) | 31 | 0.110 |
| Tocilizumab used | 49 | 0.575 |
| Rituximab used | 49 | 0.180 |
| Plasma exchanged used | 49 | 1.000 |
| **Corticosteroids used** | **49** | **0.047** |
| IVIG used | 49 | 0.687 |
| Cyclophosphamide used | 49 | 1.000 |
| Duration of hospitalization | 19 | 1.000 |
|  |  |  |
| Multivariate analysis for selected variables | *n* | Test *p*-value |
| **MRI temporal lobe abnormalities at any time** | **47** | **0.025** |
| Corticosteroids used | 47 | 0.104 |
